# Peptide Antigen Modifications Influence the On-Target and Off-Target Antibody Response for an Influenza Subunit Vaccine

**DOI:** 10.3390/vaccines13010051

**Published:** 2025-01-09

**Authors:** Megan C. Schulte, Adam C. Boll, Agustin T. Barcellona, Elida A. Lopez, Adam G. Schrum, Bret D. Ulery

**Affiliations:** 1 Department of Chemical and Biomedical Engineering, University of Missouri, Columbia, MO 65211, USA; mcsc2d@umsystem.edu (M.C.S.); atbbtf@missouri.edu (A.T.B.); schruma@health.missouri.edu (A.G.S.); 2 Department of Molecular Microbiology and Immunology, University of Missouri, Columbia, MO 65211, USA; eal6fp@missouri.edu; 3 Department of Surgery, University of Missouri, Columbia, MO 65211, USA; 4 NextGen Precision Health Institute, University of Missouri, Columbia, MO 65211, USA; 5 Materials Science & Engineering Institute, University of Missouri, Columbia, MO 65211, USA

**Keywords:** vaccine, micelle, influenza, M2, peptide amphiphile, off-target antibodies

## Abstract

Background/Objectives: Peptide amphiphile micelles (PAMs) are an exciting nanotechnology currently being studied for a variety of biomedical applications, especially for drug delivery. Specifically, PAMs can enhance in vivo trafficking, cell-targeting, and cell interactions/internalization. However, modifying peptides, as is commonly performed to induce micellization, can influence their bioactivity. In our previous work, murine antibody responses to PAMs containing the influenza antigen M2_2–16_ were slightly incongruous with prior PAM vaccine studies using other antigens. In this current work, the effect of native protein linkages and non-native micellizing moieties on M2 immunogenicity was studied. Methods: PAMs were synthesized using an elongated M2 antigen (i.e., Palm_2_K-M2_1–24_-(KE)_4_). The PAMs were characterized, then their immunogenicity was evaluated with bone marrow-derived dendritic cells and in mice. Results: Although the modification scheme yielded immunogenic PAMs, these PAMs induced a substantial amount of off-target antibody production compared to unmodified peptidyl micelles (PMs, M2_1–24_ peptide). Conclusions: While the impact PAM-induced off-target antibodies had on vaccine efficacy remains to be elucidated, on-target antibodies from both PAM- and PM-vaccinated mice were excitingly able to recognize the M2 antigen within the context of the full M2 protein. This provides preliminary evidence that the PAM-induced on-target antibodies will at minimum be able to recognize the influenza virus upon exposure.

## 1. Introduction

Peptide amphiphile micelles (PAMs) are a lipid-based nanoparticle system that can deliver bioactive peptides for immunomodulation [1,2], cancer therapy [3] and atherosclerosis theranostics [4,5]. PAMs are self-assembled structures of lipopeptides (or peptide amphiphiles, PAs) held together by the hydrophobic intermolecular association of their lipid-portion. Unlike peptides that do not self-assemble, PAMs are less susceptible to protease-mediated degradation and are able to retain defined peptide secondary structures [6,7,8]. PAMs are also able to deliver high local concentrations of peptides to cells and improve peptide-cell association [9]. In particular, micelle size and morphology can be tuned to dictate their immunogenicity, which has important implications for their use in vaccinology [10]. Favorable micelle morphology for immunostimulation has been achieved by adding a dipalmitoyllysine on the N-terminus of the antigen and a zwitterion-like charge block of alternating lysines and glutamic acids on the C-terminus (i.e., Palm_2_K-peptide-(KE)_4_), which creates small spherical and/or cylindrical micelles that are ideal for trafficking through lymphatic vessels and being taken up by antigen presenting cells [10].

Despite the aforementioned benefits, the addition of lipids and non-native residues to a bioactive peptide (e.g., in order to induce micellization) can potentially affect the functionality and bioactivity of the peptide itself. Previously, we used the M2_2–16_ antigen (a B cell antigen from the ectodomain of the influenza matrix 2 protein) to assess its potential as a universal subunit vaccine for influenza [11]. We showed that these PAMs elicited IgG titers that were comparable to the micelle-forming unmodified peptide group (i.e., peptidyl micelles; PMs), whereas, in studies using different antigens, PAMs elicited higher titers when compared to the unmodified peptide group [1,12].

In this work, we expanded the M2 antigen of focus from M2_2–16_ to M2_1–24_ (the entire external domain of the M2 protein), in order to investigate the flanking region effects on PAM immunogenicity.

Our hypothesis was that the addition of neighboring amino acids to the original M2_2–16_ antigen, would act as native linkers between the non-native modifications and the antigen producing results that better aligned with prior PAM vaccine studies using other antigens [12,13]. Like the M2_2–16_ antigen, the extended M2_1–24_ antigen has been shown to be quite conserved, thus also making it a good candidate as a universal influenza vaccine [14,15,16]. Additionally, the entire M2_1–24_ region has been used by several groups as an antigen in itself, further validating its viability as an alternative [17,18,19]. In addition to the antigen-containing peptide or PA, the adjuvant Pam_2_CSK_4_, which was previously shown to form heterogenous micelles with both M2_2–16_ peptides and the Palm_2_K-M2_2–16_-(KE)_4_ PAs and substantially increased antibody titers, was co-delivered to maximize antibody responses to the peptides and PAMs [11].

## 2. Materials and Methods

### 2.1. Peptide Synthesis and Purification

Pam_2_CSK_4_ was purchased from Invivogen (San Diego, CA, USA). Recombinant M2 protein was acquired from Abeomics (San Diego, CA, USA). All other peptides were synthesized by employing Fmoc solid-phase peptide synthesis on Sieber Amide resin with a Tetras Peptide Synthesizer (Louisville, KY, USA). Nα-Fmoc-L-amino acids were coupled to the resin (or peptide-on-resin) using a ratio of 3 equivalents of amino acid, 3 equivalents hydroxybenzotriazole (HOBt), 6 equivalents N, N-diisopropylethylamine (DIPEA), and 2.7 equivalents hexafluorophosphate benzotriazole tetramethyl uranium (HBTU) in N-methylpyrrolidone (NMP) to 1 molar equivalent resin. After coupling, 5% acetic anhydride and 7% DIPEA were added to the resin to cap any unreacted amines. Fmoc protecting groups were removed with 6% piperazine and 0.1 M HOBt in dimethylformamide (DMF). For lipidation, a non-native Fmoc-Lysine (Fmoc) was attached to the N-terminus of the peptide, followed by deprotection of both amines of the lysine and coupling of palmitic acid to both amines using the previously described coupling protocol. Peptides were cleaved from resin and deprotected using a cleavage cocktail of 2.5% each of water, phenol, triisopropylsilane, ethane-1,2-dithiol, and thioanisole in trifluoroacetic acid (TFA), followed by precipitation in diethyl ether. Peptides and PAs were purified to greater than 90% purity using LC-MS (Beckman Coulter, Brea, CA, USA) with a reverse-phase C18 column for peptides or a C4 column for PAs and a mobile phase of water, acetonitrile, and 0.1% TFA. Peptides and peptide amphiphiles eluted at the percent acetonitrile indicated in Table 1. LC-MS chromatograms of the purified compounds can be found in Figure S1. After purification, peptides and PAs were lyophilized and frozen until further use.

### 2.2. Micelle Characterization

Micelle formulations were characterized by critical micelle concentration (CMC), transmission electron microscopy (TEM), and circular dichroism (CD), as previously described [11]. To perform the CMC assay, a solution of 1 μM 1,6-diphenylhexatriene (DPH) was prepared by dissolving DPH in tetrahydrofuran, then diluting that 100-fold in deionized, distilled water (ddH_2_O), which was then diluted an additional 1000-fold in phosphate-buffered saline (PBS). A solution of 1 mM peptide or PA in water was serially diluted in the 1 μM DPH solution from 100 μM to 0.92 nM, incubated for 1 h in the dark, and then fluorescence was measured on a Cytation Biotek 5 Spectrophotometer (Santa Clara, CA, USA) at an excitation/emission of 350/428 nm. On a graph of fluorescence intensity versus peptide (or PA) concentration, CMCs were determined to be the intersection of two logarithmic regression lines roughly above and below, respectively, the concentration at which fluorescence intensity starts to increase significantly. To assess micelle morphology, peptides and PAs were imaged using TEM. Samples were diluted to 100 μM in PBS, then 5 μL were pipetted onto carbon-coated copper grids and incubated for 5 min. After wicking excess liquid from the grid with filter paper, grids were stained with Nano-Tungsten (2% methylamine tungstate) for 5 min, wicked, and imaged with a JEOL JEM-1400 Transmission Electron Microscope (Tokyo, Japan) at 15,000× and 25,000× magnification. Micelle size was determined using ImageJ Particle Size Analysis based on the TEM micrographs. Finally, CD was used to assess peptide secondary structure. Peptide and PA solutions were diluted to 250 μM in PBS, and then the CD of the solutions was measured from 190 to 250 nm with 1 nm intervals using a Jasco J-1500 Circular Dichroism Spectrophotometer (Oklahoma City, OK, USA). CD data (measured in triplicate) were fit to reference curves of poly(lysine) and poly(glutamine) to approximate α-helix, β-sheet, and random coil content.

### 2.3. Bone Marrow-Derived Dendritic Cell (BMDC) Studies

BMDCs were developed in tissue culture and tested for activation as previously described [11]. In brief, bone marrow was harvested from the femurs and tibias of 4-month-old BALB/c mice. Red blood cells were lysed using an ACK lysing buffer. Bone marrow cells were cultured in complete RPMI-1640 (i.e., 10% FBS, 100 U/mL penicillin, 100 μg/mL streptomycin, and 50 μM β-mercaptoethanol) with 20 ng/mL granulocyte-macrophage colony-stimulating factor (GM-CSF). Fresh media were added on days 3 and 8, and cells were split on day 6. After 10 days, cells were plated in untreated 24-well plates and cultured with peptides or PAs for 24 h (at dosages described in Table 2). The media supernatant was collected and frozen for later use. Cells were detached from the plates, blocked with Trustain FcX anti-mouse CD16/32 antibody (BioLegend, San Diego, CA, USA), and then stained with PE/Cyaninine7 anti-mouse CD11c (BioLegend), FITC anti-mouse CD40 (BioLegend), and APC anti-mouse MHC-II (BioLegend) to assess cell activation. Finally, cells were fixed with 4% paraformaldehyde. Cells were then analyzed using a BD LSR Fortessa Flow Cytometer (Franklin Lakes, NJ, USA) and gated using the strategy in Figure S2. To assess the cytokine content (i.e., TNF-α and IL-12/IL-23) in the media collected from the BMDCs, enzyme-linked immunosorbent assays (ELISAs) were completed using the protocols provided in the kits (BioLegend).

### 2.4. Vaccination Schedule

In vivo studies were conducted in accordance with protocol 32,204, as approved by the Animal Care and Use Committee of the University of Missouri. BALB/c mice (4 females and 3–4 males per group) approximately 10 weeks in age were subcutaneously injected in the nape of the neck with the vaccine formulations. Vaccines consisted of 100 μL PBS containing 20 nmol peptide or PA with or without 2.22 nmol Pam_2_CSK_4_ (Table 3). Vaccinations were administered on days 0 and 21 with blood collection on day 35. Blood was collected by cardiac puncture (after CO_2_ asphyxiation), according to ACUC guidelines.

### 2.5. Serum Enzyme-Linked Immunosorbent Assay (ELISA)

The serum was collected by centrifuging blood at 9400× *g* for 10 min and harvesting the supernatant, after which it was frozen at −80 °C until further use. ELISAs were conducted to quantify antibody content in the serum. To coat the plates, 1.69 μM of the coating peptide in a carbonate buffer was incubated in the wells at 4°C overnight. Plates were washed with 0.05% Tween-20 in PBS and then blocked with 10% FBS in PBS for 1 h. The serum was then added to wells at an initial 100× dilution, then serially diluted in assay diluent across the plate 21 times and serially diluted across the plate before being incubated at 4°C overnight. The next day, plates were washed, incubated with a 1:3000 dilution of goat anti-mouse IgG-HRP (Invitrogen, Waltham, MA, USA) for 1 h, washed again, then incubated with TMB (3,3′,5,5′ tetramethylbenzidine, BioLegend). After 30 min, the absorbance of the wells was measured at 650 nm using a Biotek Cytation 5 Spectrophotometer. The samples were normalized across plates by subtracting the absorbance of assay-diluent only wells from the absorbance of each serum-containing well. Antibody titers were calculated as the lowest serum dilution with an absorbance at least twice the absorbance of the serum samples from PBS-vaccinated mice at a given serum dilution. For the M2 protein ELISA study, the M2 protein and M2_1–24_ peptide were coated on the plates at a concentration of 0.09 μM in a carbonate buffer after which the rest of the protocol remained unchanged.

### 2.6. Statistics

One-way analysis of variance (ANOVA) and Tukey’s Honestly Significant Difference (HSD) tests were performed using GraphPad Prism software (Version 7.02). Within a graph, groups that are labeled with the same letter have similar means (*p* > 0.05), whereas pairwise comparisons without the same letter possess statically significantly different means (*p* ≤ 0.05).

## 3. Results and Discussion

### 3.1. M2_1–24_ and Palm_2_K-M2_1–24_-(KE)_4_ Formed Micelles with Vaccine Favorable Characteristics

**Figure 1:**
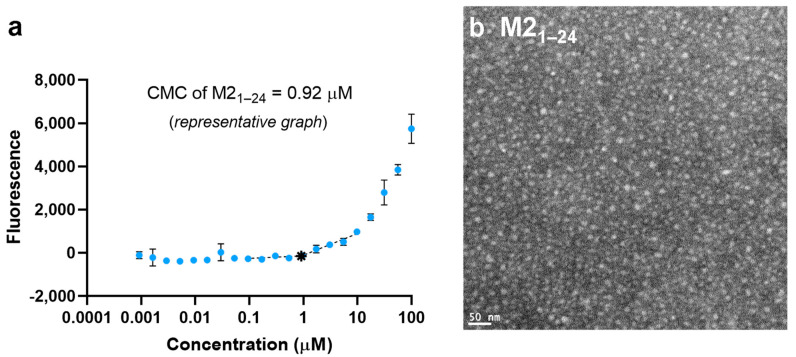

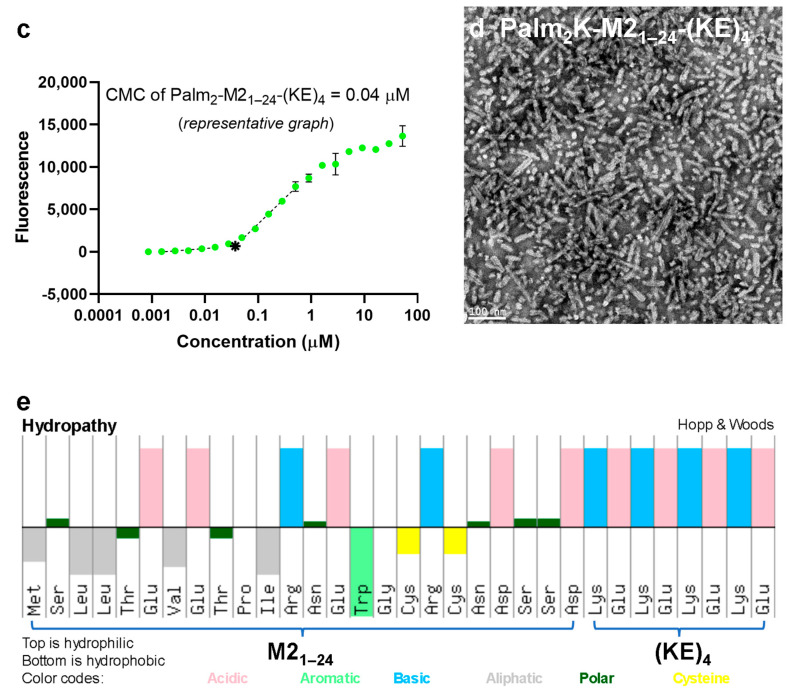
Both M2_1–24_ and Palm_2_K-M2_1–24_-(KE)_4_ self-assemble into small micelles with low critical micelle concentrations. (**a**) A representative graph of M2_1–24_ shows a CMC of 0.92 μM, denoted by ∗. (**b**) A TEM micrograph of M2_1–24_ indicates the presence of small spherical micelles. (**c**) A representative graph of Palm_2_K-M2_1–24_-(KE)_4_ shows a CMC of 0.04 μM, denoted by ∗. (**d**) A TEM micrograph of Palm_2_K-M2_1–24_-(KE)_4_ indicates the presence of spherical and short cylindrical micelles. (**e**) The hydropathy chart of M2_1–24_-(KE)_4_ (dipalmitoyllysine not shown) shows the hydrophobic and hydrophilic amino acids are interspersed throughout M2_1–24_, whereas the (KE)_4_ region imparts the strong hydrophilicity to the C-terminus of Palm_2_K-M2_1–24_-(KE)_4_ [25].

Peptides and PAs were synthesized on-resin and purified by LC-MS. The M2_1–24_ peptide and Palm_2_K-M2_1–24_-(KE)_4_ PA were characterized for their ability to micellize at sufficiently low concentrations and form small micelles that are favorable for trafficking and cell uptake. The CMC of the M2_1–24_ peptide was 1.2 ± 0.9 μM (Figure 1a), and TEM micrographs showed that the M2_1–24_ peptides formed small spherical micelles (Figure 1b), which will be referred to as peptidyl micelles (PMs). M2_1–24_ PMs had an estimated average maximum caliper diameter of 28 ± 15 nm, based on particle analysis using ImageJ (Figure S3a). Micellization was also confirmed for the Palm_2_K-M2_1–24_-(KE)_4_ PA. The CMC of Palm_2_K-M2_1–24_-(KE)_4_ was 0.038 ± 0.018 μM (Figure 1c), which was sufficiently low to ensure micellization in vivo, even when considering their dissemination into lymphatic vessels or more diffusely in the blood for which an estimated 1.5–2 mL total volume per mouse was used [20]. In the Palm_2_K-M2_1–24_-(KE)_4_ PAMs, the strong hydrophobic contribution of the dipalmitoyllysine probably caused the lower CMC (relative to the unmodified M2_1–24_ PMs) [21,22,23]. Micrographs from TEM (Figure 1d) showed that Palm_2_K-M2_1–24_-(KE)_4_ formed a combination of spherical and short cylindrical micelles, which have been shown to be ideal for trafficking to draining lymph nodes and uptake by antigen-presenting cells [10]. Employing ImageJ to perform particle analysis, Palm_2_K-M2_1–24_-(KE)_4_ PAMs were found to have an estimated average maximum caliper diameter of 37 ± 24 nm (Figure S3b).

The differences in micelle morphology between the unmodified M2_1–24_ PMs and the Palm_2_K-M2_1–24_-(KE)_4_ PAMs can potentially be explained by a difference in the hydrophobicity/amphipathicity between the micelle types [24]. The PAMs had strong amphipathicity caused by the dipalmitoyllysine on the N-terminus and the set of charged residues on the C-terminus (Figure 1e), which would allow for the PAs in the micelles to have a lipid core and a corona consisting of the (KE)_4_ charge block, with the M2_1–24_ sequence most likely between the two. This relatively linear conformation of the PA in the PAMs could facilitate tight packing at a low concentration, leading to the low CMC and the development of cylindrical micelles (in which individual PAs have tighter packing angles) than in spheres (as was found for the M2_1–24_ PMs) [24].

**Figure 2:**
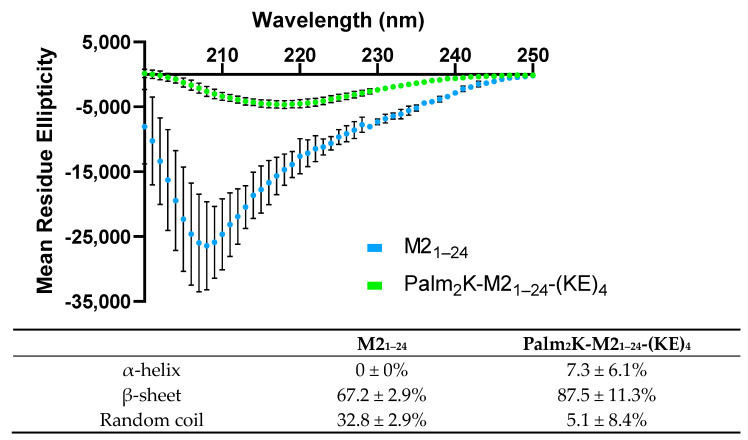
Circular dichroism of M2_1–24_ and Palm_2_K-M2_1–24_-(KE)_4_ shows substantial differences between peptides and peptide amphiphiles. Circular dichroism spectra show that M2_1–24_ was predominantly β-sheet, although with significant random coil character. Palm_2_K-M2_1–24_-(KE)_4_ was almost entirely β-sheet with minimal amounts of α-helix and random coil.

To further characterize the micelles, CD was conducted to evaluate the secondary structure of M2_1–24_ and the peptide content within Palm_2_K-M2_1–24_-(KE)_4_ (Figure 2). M2_1–24_ PMs had substantial random coil content (32%) but were still predominantly β-sheet (67%). Palm_2_K-M2_1–24_-(KE)_4_ PAMs were nearly entirely β-sheet (87%) with the rest split between α-helix and random coil. The β-sheet-dominant structure of the PAMs aligns well with what has been observed for other PAMs using the Palm_2_K-peptide-(KE)_4_ configuration, including in Palm_2_K-M2_2–16_-(KE)_4_ (97% β-sheet), Palm_2_K-OVA_BT_-(KE)_4_ (91% β-sheet), and Palm_2_K-VIP-(KE)_4_ (67% β-sheet) [2].

### 3.2. Bone Marrow-Derived Dendritic Cell Activation Was Driven by the Adjuvant Pam_2_CSK_4_

**Figure 3:**
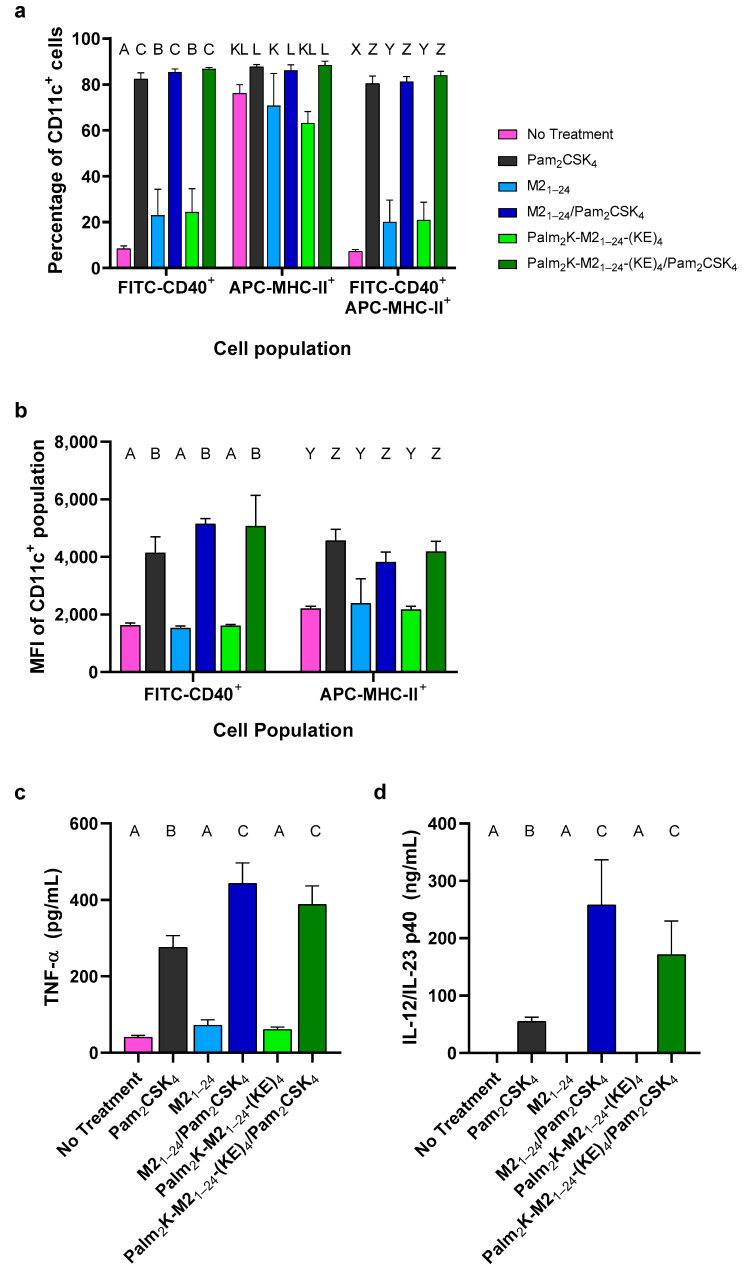
BMDC activation was largely driven by treatment with Pam_2_CSK_4_, with pro-inflammatory cytokine secretion further enhanced by PM- or PAM-mediated delivery of Pam_2_CSK_4_. (**a**) The percentage of cells expressing substantial amounts of CD40 was significantly increased in cells treated with Pam_2_CSK_4_-containing formulations. There was not an appreciable increase in MHC-II expression. (**b**) The MFIs in the FITC and APC channels were significantly elevated among CD11c^+^CD40^+^ and CD11c^+^MHC-II^+^ cells, respectively, for those treated with Pam_2_CSK_4_. Treatment with PMs or PAMs alone did not have a stimulatory effect. (**c**) TNF-α and (**d**) IL-12/IL-23 secretion was above baseline for all Pam_2_CSK_4_-containing groups. Interestingly, micelles alone did not induce pro-inflammatory cytokine secretion, but micelle-mediated adjuvant delivery did enhance this effect above Pam_2_CSK_4_ alone. Within a graph, groups that possess different letters have a statistically significant difference in mean (*p* ≤ 0.05) whereas those that possess the same letter have similar means (*p* > 0.05). Only treatment groups within the same channel and cell population were compared to each other.

Because both M2_1–24_ peptide and Palm_2_K-M2_1–24_-(KE)_4_ PA produced micelles with favorable properties, the immunogenicity of the PMs and PAMs was evaluated with BMDCs. Dendritic cells (DCs) play a significant role in antigen presentation and activation of CD4+ T cells, which triggers downstream effects on B cells and antibody production, highlighting the importance of DCs in vaccine-induced immune responses [26,27,28]. BMDCs were processed for flow cytometry and identified by CD11c expression, whereas the markers CD40 and MHC-II were used as markers of BMDC stimulation [29,30]. BMDC activation was primarily dictated by the presence or absence of the TLR2 agonist Pam_2_CSK_4_, with some minor influence by the presence of micelles, as seen in Figure 3. Treatment with M2_1–24_ PMs or Palm_2_K-M2_1–24_-(KE)_4_ PAMs resulted in a slight increase in the percentage of CD11c^+^ cells expressing CD40 (Figure 3a). There was a much greater increase observed when the adjuvant Pam_2_CSK_4_ was added to treatment, dwarfing the influence of micelle presence. There was no treatment effect on MHC-II expression of CD11c^+^ cells compared to untreated cells. The median fluorescence intensity (MFI) in the FITC (CD40) and APC (MHC-II) channels of CD11c^+^CD40^+^ and even CD11c^+^MHC-II^+^ populations, respectively, was heightened in response to treatment with an adjuvant, but not due to exposure to PMs or PAM alone (Figure 3b). Clearly, all treatment groups containing Pam_2_CSK_4_ activated the BMDCs, based on CD40 expression. The less pronounced differences in MHC-II expression between treatment groups were likely due to MHC-II being constitutively expressed on most BMDCs, even in the basal state of the assay observed in Figure 3a. Also, previous research like that from Dearman and colleagues has found a less pronounced increase in MHC-II expression above baseline compared to other markers, including CD40, CD80, and CD86 in BMDCs treated with lipopolysaccharide (LPS), in accordance with our observations in Figure 3b [30].

To evaluate the secretion of pro-inflammatory cytokines, TNF-α, and IL-12/IL-23, sandwich ELISAs were conducted using media collected from the treated BMDCs. Specifically, IL-12/IL-23 was quantified using an ELISA specific for the p40 monomer, which is present in both IL-12 and IL-23 heterodimers and has been frequently used to assess BMDC activation [30,31,32,33]. As with the upregulation of activation markers, the elevated secretion of TNF-α and IL-12/IL-23 p40 was largely driven by the presence of Pam_2_CSK_4_ (Figure 3c,d). It was not surprising that the primary effect on pro-inflammatory cytokine secretion was the adjuvant, as TNF-α, IL-12, and IL-23 expression have all been shown to be elevated in activated BMDCs upon initiation of the MyD88-NF-κB pathway, which is triggered by Pam_2_CSK_4_ via TLR2 [31,34,35,36,37]. Beyond the adjuvant effect on cytokine secretion, there was a further statistically significant increase in TNF-α and IL-12/IL-23 p40 secretion in both adjuvant-loaded micelle treatment groups (i.e., M2_1–24_/Pam_2_CSK_4_ and Palm_2_K-M2_1–24_-(KE)_4_/Pam_2_CSK_4_), suggesting an additional effect of co-delivery that was not observed with CD40 nor MHC-II expression. Like Pam_2_CSK_4_, Palm_2_K-M2_1–24_-(KE)_4_ has two 16-carbon lipids at its N-terminus, which previous studies have shown can effectively stimulate TLR2 [38]. However, these lipids alone are not sufficient for TLR2 activation, which could explain the less consistent immunogenic effect of PMs and PAMs delivered alone compared to when Pam_2_CSK_4_ is present [12,39,40].

### 3.3. Peptide Amphiphile Micelles Induced On- and Off-Target Antibody Production

**Figure 4:**
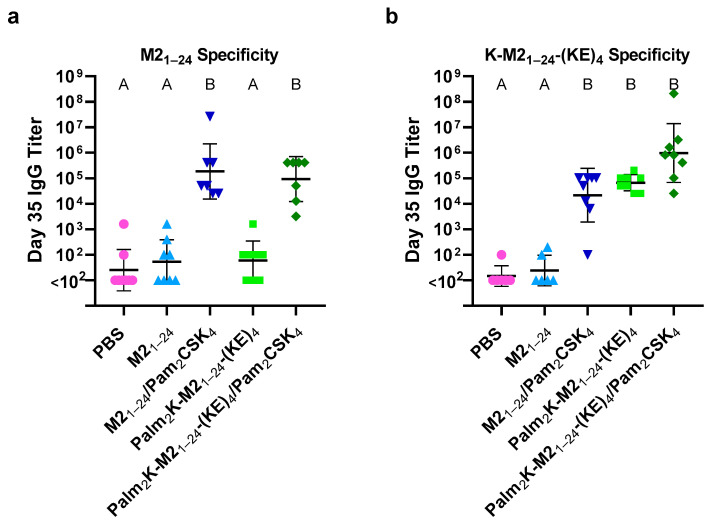
Day 35 IgG Titers against M2_1–24_ and K-M2_1–24_-(KE)_4_ peptide coatings showed PAMs induced on-target and off-target antibody production. (**a**) Vaccine groups with Pam_2_CSK_4_ produced similar, high IgG titers against the M2_1–24_ coating antigen whereas those without Pam_2_CSK_4_ did not produce appreciable titers above baseline. (**b**) K-M2_1–24_-(KE)_4_-specific titers in both PAM groups were elevated relative to their respective PM groups, though only statistically significant differences were observed for the adjuvant-free groups. Within a graph, groups that possess different letters have a statistically significant difference in mean (*p* ≤ 0.05), whereas those that possess the same letter have similar means (*p* > 0.05).

To investigate the immunogenicity of the micelles in vivo, mice were vaccinated with the M2_1–24_ PM or Palm_2_K-M2_1–24_-(KE)_4_ PAM formulations. The peptides and vaccine dosages used in this study are outlined in Table 3. The serum collected from the mice was used to assess antibody titers by ELISA. IgG titers were first tested using the M2_1–24_ peptide as the plate coating, as that most closely represented the antigen within the context of the influenza virus. Again, the presence of adjuvant was found to be the primary driving force in inducing a productive immune response, with both PM and PAMs eliciting high IgG titers when co-delivered with Pam_2_CSK_4_ (Figure 4a). While there were more responders in mice vaccinated with only PMs or PAMs when compared to mice vaccinated with PBS, there were no statistical differences found between these groups. These results aligned with previous results using the M2_2–16_ antigen, in which Palm_2_K-M2_2–16_-(KE)_4_ PAMs did not produce titers above the unmodified M2_2–16_ peptides, which is an effect that had not been observed with other antigens [1,11,12]. To investigate the cause of these results, another ELISA was conducted using K-M2_1–24_-(KE)_4_ as the plate coating, which would capture antibody specificity against the entire peptidyl component of the PA. ELISAs completed using this new coating antigen produced an interesting result in that the IgG titers of both PAM-containing vaccine groups (i.e., Palm_2_K-M2_1–24_-(KE)_4_ and Palm_2_K-M2_1–24_-(KE)_4_/Pam_2_CSK_4_) increased considerably whereas the titers of the PM-containing groups (i.e., M2_1–24_ and M2_1–24_/Pam_2_CSK_4_) stayed relatively similar to what was seen with the M2_1–24_ coating (Figure 4b). This result showed that overall IgG titers elicited by the PAMs actually were higher than by the PMs (especially when comparing between groups without adjuvant), which was more in line with what had been previously observed using other antigens like J8 and OVA_BT_ [1,12]. A likely explanation for the difference in results between the M2_1–24_ and K-M2_1–24_-(KE)_4_ ELISAs was that the PAMs were eliciting the production of off-target antibodies that were not entirely specific to the M2_1–24_ region, but to the modifications on the N- and/or C-terminus of the PA.

**Figure 5:**
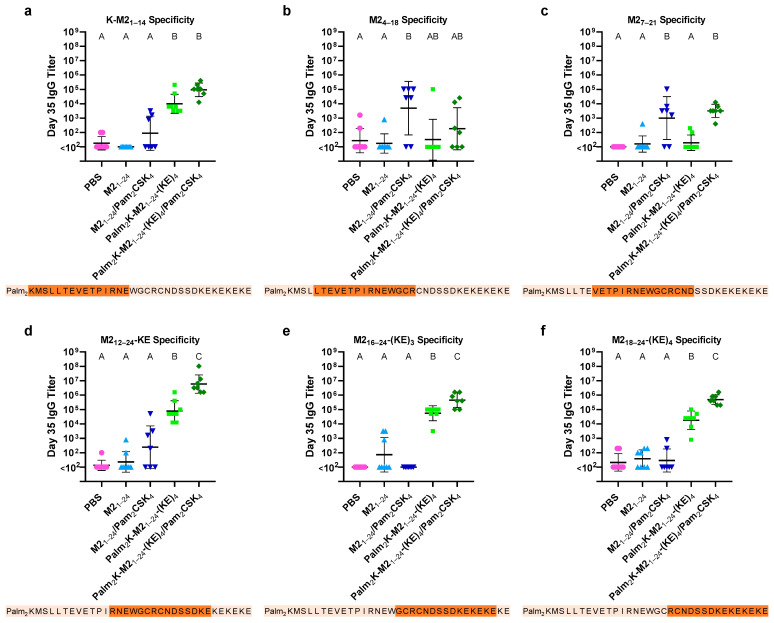
Peptide walk ELISA showed that PAM-vaccinated mice produced higher titers when the non-native amino acids of the PA were present. (**a**) The PAM groups elicited higher titers against the coating antigen K-M2_1–14_ than the PM groups. (**b**) M2_1–24_/Pam_2_CSK_4_ produced the highest titer against M2_4–18_, although not statistically significantly higher than the PAM groups. (**c**) The titers against M2_7–21_ were highest in groups with Pam_2_CSK_4_. (**d**) The PAM groups, (especially with Pam_2_CSK_4_), elicited the highest titers against M2_12–24_-KE. (**e**) The PM groups produced almost no titers against M2_16–24_-(KE)_3_, while PAM titers were several orders of magnitude higher. (**f**) PAM groups elicited high titers against M2_18–24_-(KE)_4_, while PM group titers were at baseline. The darker orange letters underneath the graphs indicate the area of the coating antigen within the context of the entire peptide amphiphile Palm_2_K-M2_1–24_-(KE)_4_ sequence. Within a graph, groups that possess different letters have a statistically significant difference in mean (*p* ≤ 0.05) whereas those that possess the same letter have similar means (*p* > 0.05).

To investigate this off-target antibody production effect further, a series of “peptide walk” ELISAs were conducted in order to elucidate the sequence specificity of the antibodies in the vaccinated mice. A series of 15-mer peptides were synthesized from K-M2_1–14_ to M2_18–24_-(KE)_4_, shifting 2–5 amino acids towards the C-terminus with each peptide. These peptides were used individually as the coating antigen in an ELISA to test antibody specificity from day 35 serum (Figure 5). Mice vaccinated with Palm_2_K-M2_1–24_-(KE)_4_ PAMs (with and without Pam_2_CSK_4_) mice had higher titers than M2_1–24_ PM groups (with and without Pam_2_CSK_4_) against the coating antigens of K-M2_1–14_ (Figure 5a), M2_12–24_-KE (Figure 5d), M2_16–24_-(KE)_3_ (Figure 5e), and M2_18–24_-(KE)_4_ (Figure 5f) (i.e., all coating antigens containing non-native residues on the N- or C-terminus). The fact that PAMs elicited higher titers against both the N-terminal and C-terminal portions of the PA suggests a polyclonal antibody response. The especially high titers in Figure 5e,f provides strong evidence for off-target antibody production towards the C-terminus. Titers were most similar when comparing the coating antigens M2_7–21_ (Figure 5c) and M2_1–24_ (Figure 4a), while, interestingly, the titers of the PAM groups against M2_4–18_ (Figure 5b) were substantially lower than the PM groups. The stark increase in relative PAM titers against the K-M2_1–14_, and even M2_1–24_, coating antigen compared to those against the M2_4–18_ coating antigen suggests that at least one of the first three amino acids of the M2_1–24_ antigen was critical for binding of PAM vaccine-induced antibodies. The fact that Palm_2_K-M2_1–24_-(KE)_4_/Pam_2_CSK_4_ titers against M2_4–18_ were low, despite previous work showing good immunogenicity of Palm_2_K-M2_2–16_-(KE)_4_/Pam_2_CSK_4_ PAMs against M2_1–24_, further suggests the importance of the second and/or third residues in M2 for PAM-elicited antibody binding [11].

The off-target antibody responses were unexpected given that the (KE)_4_ motif is found in multiple places throughout the murine proteome as determined by a peptide sequence search of the UniProt database, which would suggest that (KE)_4_ should be non-immunogenic [41]. Yet, there was a clear antibody response against (KE)_4_ shown here. There is some evidence in the literature of peptides that can exert an adjuvanting effect when paired with an antigenic peptide, even without being inherently immunogenic themselves (i.e., KFE8 and Q11) [42,43], which can likely be at least partially attributed to the self-assembly driven by these peptides. Given that the production of off-target antibodies seen here has not been previously observed in PAMs using other antigens and that (KE)_4_ should be non-immunogenic, it is possible that the M2_1–24_ sequence itself was acting as an adjuvant for the (KE)_4_ region of the PA. Further testing will be required to investigate this hypothesis; however, there are some exciting implications that could result from this. Namely, that the (KE)_4_ moiety could be replaced with a biologically relevant peptide, such as a CD8 or CD4 antigen to further expand the immunogenicity of the vaccine.

### 3.4. Antibodies Elicited by PMs and PAMs Recognized M2_1–24_ Within the Full M2 Protein

**Figure 6:**
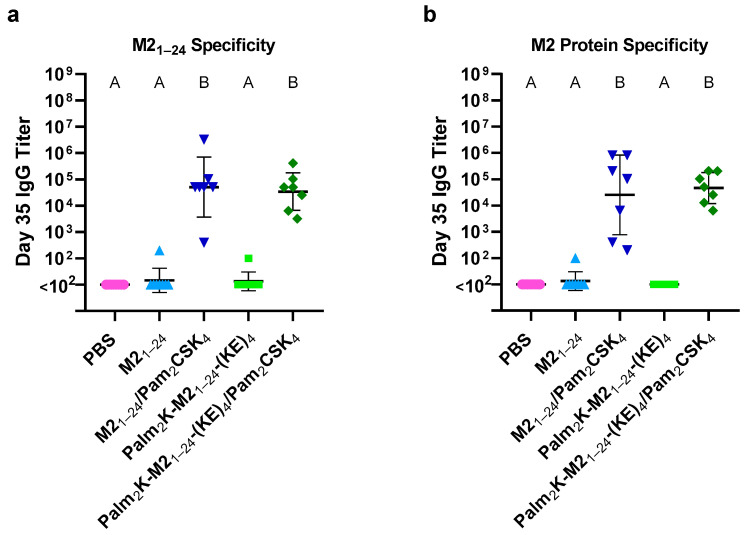
Both peptidyl micelles and peptide amphiphile micelles were able to induce the production of antibodies capable of recognizing the M2 protein. To better compare results between titers against M2_1–24_ and M2 protein, a reduced coating concentration of 0.09 μM was used for both coating antigens. (**a**) Titers were most influenced by the presence of Pam_2_CSK_4_ in the vaccine regardless of micelle type. (**b**) M2 protein-specific IgG titers were again more influenced by the presence of Pam_2_CSK_4_ in the micelle vaccine formulation. Titers, spread, and number of responders aligned well between the peptide and protein coatings. Within a graph, groups that possess different letters have a statistically significant difference in mean (*p* ≤ 0.05), whereas those that possess the same letter have similar means (*p* > 0.05).

To assess whether the off-target antibody production in the PAM-vaccinated mice affected the ability of the antibodies to recognize the M2_1–24_ epitope as a part of the entire M2 protein, an ELISA was conducted using the entire M2 protein as the plate coating. This was compared to an ELISA performed again using the M2_1–24_ peptide, but at a lower coating concentration for a more accurate comparison of the results between the different plate coatings (Figure 6). Another notable result was that in the context of the M2 protein ELISA (Figure 6) especially, the range of antibody titers for the adjuvanted Palm_2_K-M2_1–24_-(KE)_4_ PAM-vaccinated group was quite a bit narrower than that of the adjuvanted PM vaccinated-group (M2_1–24_/Pam_2_CSK_4_). A potential explanation for this result is that the PAMs were likely more ordered (and have a more consistent antigen display) than the PMs because of the stronger hydrophobic association effect conveyed by the lipids in the PAMs. It is also worth noting that although there was evidently off-target antibody production caused by the PAMs, there was still a comparable M2 protein-specific titer between the PAMs and PMs. If in a future study off-target antibody production can be prevented in the PAMs, the on-target titers in PAM-vaccinated mice could possibly be higher than in the PM-vaccinated mice, as was observed in studies employing PAMs with other antigens (i.e., J8 and OVA_BT_) [1,12].

## 4. Conclusions

PMs and PAMs from the M2_1–24_ antigen were generated that were of sufficiently small size and low enough CMCs to be good candidates as vaccine delivery vehicles. Both M2_1–24_ PMs and Palm_2_K-M2_1–24_-(KE)_4_ PAMs, (with the aid of an adjuvant), elicited strong antibody titers specific to the M2_1–24_ antigen in vivo. However, upon further investigation, it became apparent that there was considerable off-target antibody production in the PAM-vaccinated mice, especially against the non-native lysine on the N-terminus and the (KE)_4_ region on the C-terminus of the antigen. Unfortunately, the addition of native residues to both sides of the M2_2–16_ antigen (one amino acid on the N-terminus and eight amino acids on the C-terminus) was insufficient to prevent the production of off-target antibodies. This information, and the fact that this phenomenon was not seen in studies of other epitopes, suggested an antigen-specific effect, specifically, that the M2_1–24_ antigen was able to exert an adjuvanting effect on the (KE)_4_ peptide moiety. It remains to be seen whether this off-target antibody production has any immune distraction or synergistic effects in terms of vaccine efficacy, but this proposed adjuvanting effect could have exciting implications for linking multiple antigens in a single PA. Regardless, it was promising that antibodies from PM- and PAM-vaccinated mice were still able to recognize their target antigen in the context of the entire M2 protein. Future work to advance micelle technology for influenza vaccine applications will compare the immune responses elicited by PMs and PAMs against publicly available influenza vaccines.

## Figures and Tables

**Table 1:** Percent Acetonitrile at Peptide and Peptide Amphiphile Elution Time.

Peptide	Approximate % Acetonitrile at Elution
M2_1–24_	30%
K-M2_1–24_-(KE)_4_	25%
Palm_2_K-M2_1–24_-(KE)_4_	60%
K-M2_1–14_	25%
M2_4–18_	25%
M2_7–21_	25%
M2_12–24_-KE	15%
M2_16–24_-(KE)_3_	10%
M2_18–24_-(KE)_4_	10%

Where M2_1–24_ is MSLLTEVETPIRNEWGCRCNDSSD, subscripts after M2 denote the amino acid positions within the M2 ectodomain, Palm_2_K is dipalmitoyllysine, and (KE)_x_ is a repeat of lysine-glutamic acid for a total of x number of times.

**Table 2:** Vaccine Formulations Used in BMDC Activation Assessment.

Vaccine Group	Vaccine Dosage
No Treatment	No Peptide nor PA
Pam_2_CSK_4_	0.2 μM Pam_2_CSK_4_
M2_1–24_	1.8 μM M2_1–24_
M2_1–24_/Pam_2_CSK_4_	1.8 μM M2_1–24_ and 0.2 μM Pam_2_CSK_4_
Palm_2_K-M2_1–24_-(KE)_4_	1.8 μM Palm_2_K-M2_1–24_-(KE)_4_
Palm_2_K-M2_1–24_-(KE)_4_/Pam_2_CSK_4_	1.8 μM Palm_2_K-M2_1–24_-(KE)_4_ and 0.2 μM Pam_2_CSK_4_

**Table 3:** Vaccine formulations used for the in vivo immunization study.

Vaccine Group	Vaccine Dosage
PBS	No Peptide nor PA
M2_1–24_	20 nmol M2_1–24_
M2_1–24_/Pam_2_CSK_4_	20 nmol M2_1–24_ and 2.22 nmol Pam_2_CSK_4_
Palm_2_K-M2_1–24_-(KE)_4_	20 nmol Palm_2_K-M2_1–24_-(KE)_4_
Palm_2_K-M2_1–24_-(KE)_4_/Pam_2_CSK_4_	20 nmol Palm_2_K-M2_1–24_-(KE)_4_ and 2.22 nmol Pam_2_CSK_4_

## Data Availability

All data are contained within the article and Supplementary Materials.

## Supplementary Materials

The following supporting information can be downloaded at: https://www.mdpi.com/article/10.3390/vaccines13010051/s1, Figure S1: Peptides were purified to greater than 90% purity using high performance liquid chromatography-mass spectrometry (LC-MS); Figure S2: Bone-marrow derived dendritic cells were gated using the above strategy; Figure S3: ImageJ was used to analyze micelle size.
