# Supplementary material for: Peptide Antigen Modifications Influence the On-Target and Off-Target Antibody Response for an Influenza Subunit Vaccine
Source: Vaccines (Basel). 2025 Jan 9;13(1):51. doi: 10.3390/vaccines13010051 (PMC11768957; doi:10.3390/vaccines13010051)
Supplement: Supplementary file 1 [file vaccines-13-00051-s001.zip › vaccines-3257431-supplementary.pdf]

Supplementary Information

a M2$_{1-24}$

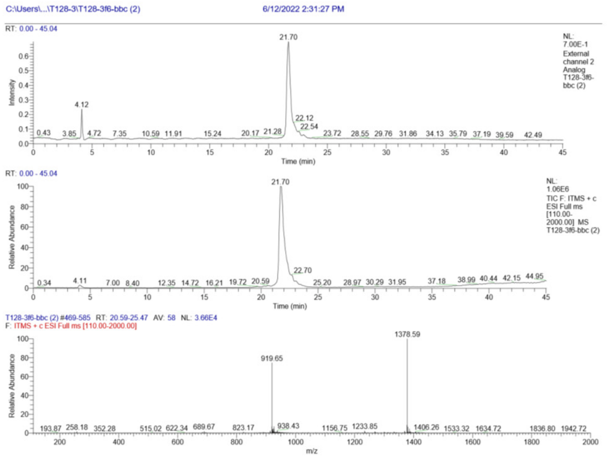

b K-M2$_{1-24}$-(KE)$_4$

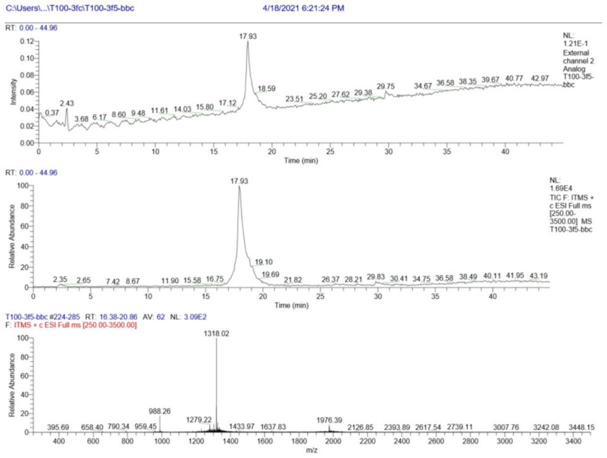

c Palm$_2$K-M2$_{1-24}$-(KE)$_4$

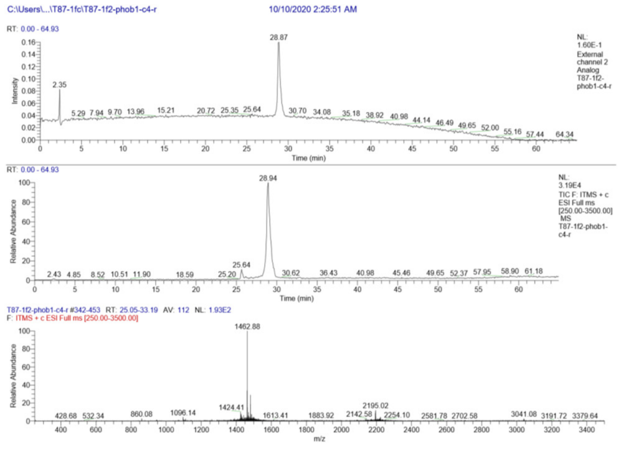

d K-M$2_{1–14}$

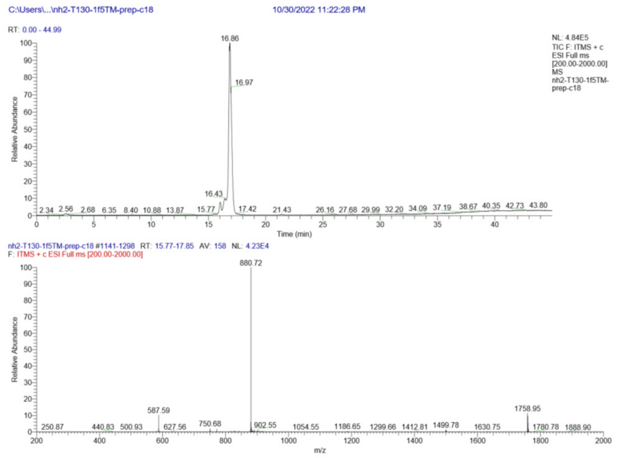

e M2_{4-18}

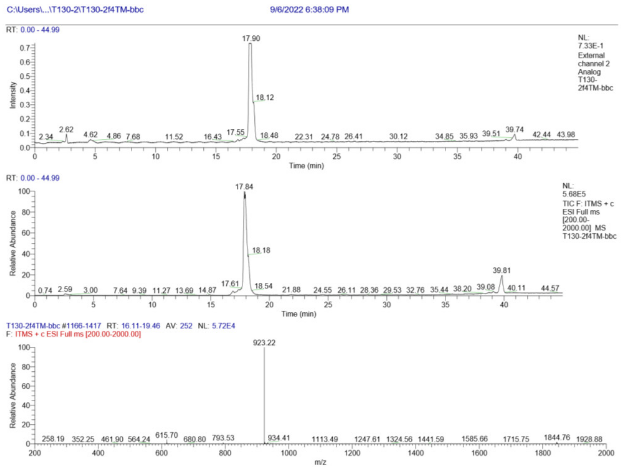

f M2_{7-21}

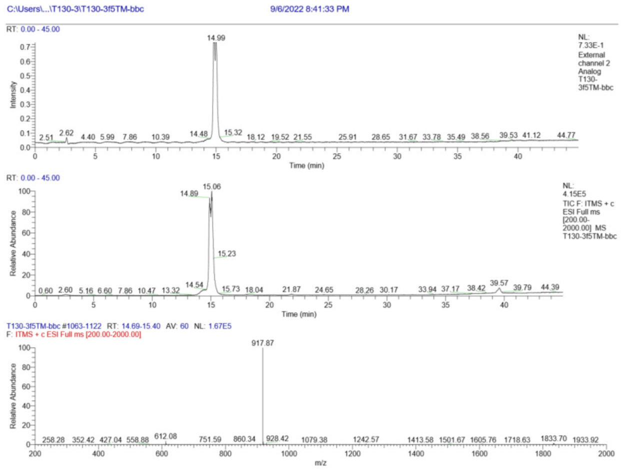

g M2$_{12–24}$-KE

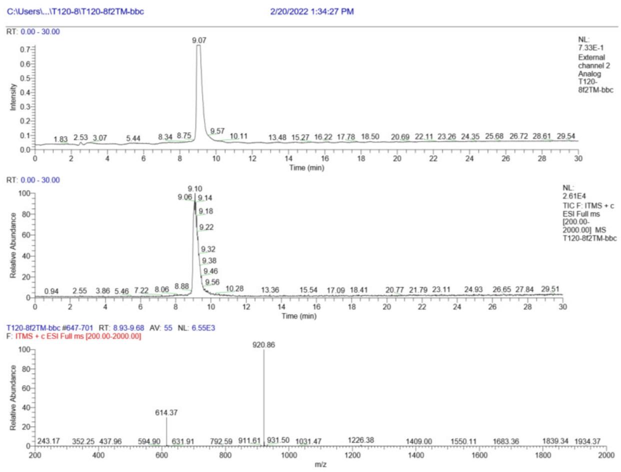

h M2$_{16-24}$-(KE)$_3$

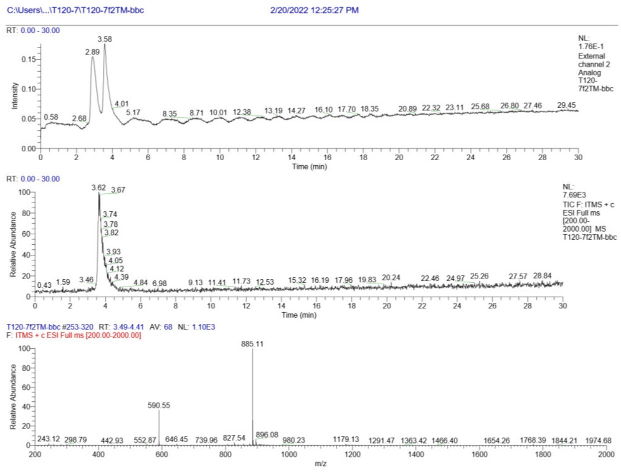

i M2$_{18-24}$-(KE)$_4$

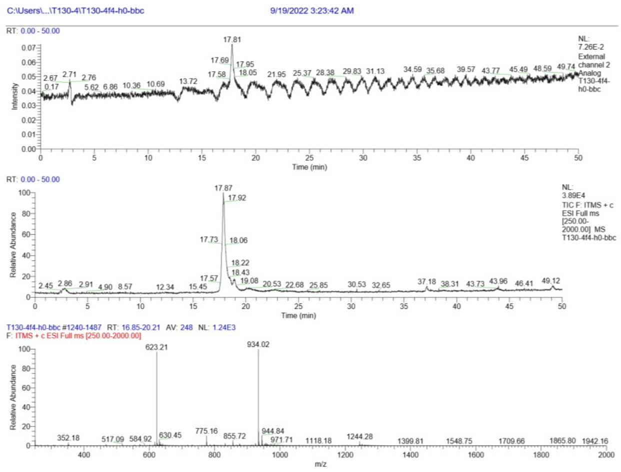

Figure S1. Peptides were purified to greater than 90% purity using high performance liquid chromatography-mass spectrometry (LC-MS). LC-MS analyses are shown for purified (a) $M2_{1–24}$, (b) K-$M2_{1–24}$-$(KE)_4$, (c) $Palm_2$K-$M2_{1–24}$-$(KE)_4$, (d) K-$M2_{1–14}$, (e) $M2_{4–18}$, (f) $M2_{7–21}$, (g) $M2_{12–24}$-KE, (h) $M2_{16–24}$-$(KE)_3$, and (i) $M2_{18–24}$-$(KE)_4$. For each peptide or PA, panels from top to bottom show the UV chromatogram (where applicable), total ion chromatogram, and mass spectrum.

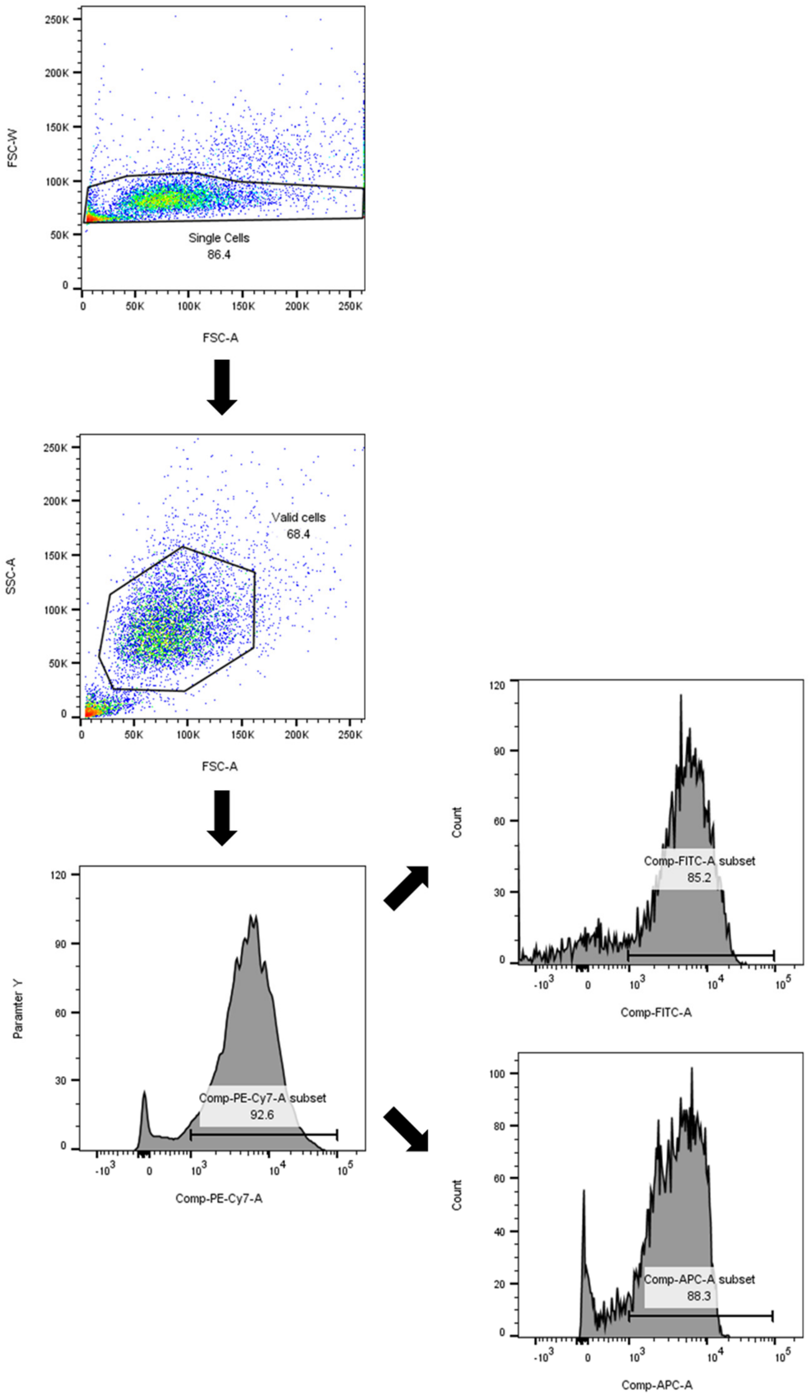

Figure S2. Bone-marrow derived dendritic cells were gated using the above strategy. Debris and aggregates were removed from the population by gating FSC-W versus FSC-A, then SSC-A versus FSC-A. Dendritic cells were identified as cells with PE-Cy7 fluorescence intensities above 1000, where PE-Cy7 fluorescence indicated CD11c expression. Activated dendritic cells were identified as CD11c^{+} cells with FITC and/or APC fluorescence intensities greater than 1000, where FITC and APC fluorescence indicated CD40 and MHC-II expression, respectively.

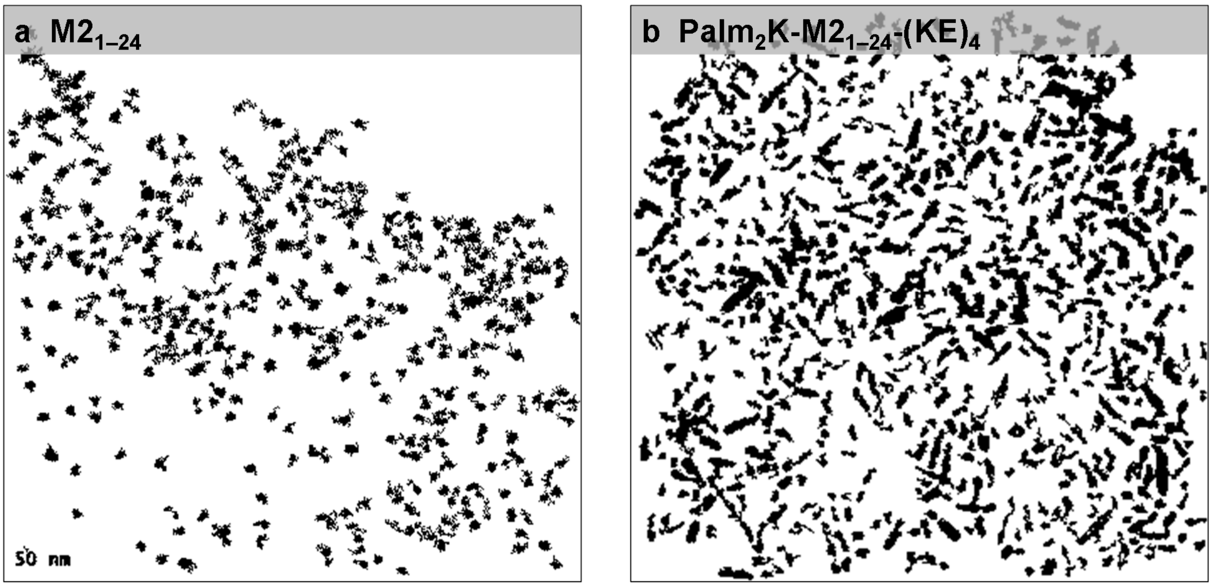

Figure S3. ImageJ was used to analyze micelle size. Using the particle analysis feature, particle masks were created from which size information could be obtained. (a) M2$_{1\text{-}24}$ PMs (239 micelles analyzed) had a range of 14 - 125 nm with an average maximum caliper diameter of 28 ± 15 nm. (b) Palm$_2$K-M2$_{1\text{-}24}$-(KE)$_4$ PAMs (422 micelles analyzed) had a range of 14 - 162 nm with an average maximum caliper diameter of 37 ± 24 nm.
